# Perception of virus effects and vaccination among people with multiple sclerosis in Saudi Arabia

**DOI:** 10.3389/fneur.2026.1816182

**Published:** 2026-10-12

**Authors:** Hind Alnajashi, Khadijah Ibraheem Basheikh, Bushra Obaidallah A. Alqurayqiri, Fayzah Shaikh Albar, Shahad Ayed M. Algarni, Lana Saeed Allakhmi

**Affiliations:** 1 Department of Neurology, Faculty of Medicine, King Abdulaziz University, Jeddah, Saudi Arabia; 2 Faculty of Medicine, King Abdulaziz University, Jeddah, Saudi Arabia

**Keywords:** counseling, disease-modifying therapies, Epstein–Barr virus, influenza vaccine, multiple sclerosis, vaccination, vaccine hesitancy

## Abstract

**Background:**

People with multiple sclerosis (pwMS) are at increased risk of infection due to immune dysregulation and exposure to disease-modifying therapies (DMTs). Despite this risk, real-world vaccine uptake remains inconsistent. This study evaluated vaccination readiness, counseling practices, and infection-related perceptions among pwMS in Saudi Arabia.

**Methods:**

A cross-sectional survey was conducted among adults with MS (*n* = 204) recruited using non-probability (convenience) sampling from tertiary-care clinical and community-based sources. The survey assessed demographic characteristics, vaccination attitudes and uptake, counseling experiences, perceptions of infection risk related to MS and DMTs, and awareness of Epstein–Barr virus (EBV). Internal consistency of multi-item measures was acceptable (Cronbach's α = 0.72). Descriptive statistics were used to summarize outcomes.

**Results:**

The cohort was relatively young (mean age 35.7 years), with two-thirds aged 25–44 years (25–34 years: 38.2%; 35–44 years: 36.8%). Most participants held a bachelor's degree (66.7%). Attitudes toward routine vaccination were frequently non-committal: 47.6% were somewhat or very likely to vaccinate, 33.8% were neutral, and 18.6% were somewhat or very unlikely; separately, 60.8% agreed or somewhat agreed that routine vaccines might be harmful for pwMS. COVID-19 vaccination uptake was high (72.5%), whereas influenza vaccination was low (30.9%). Uptake of other adult vaccines, including hepatitis B, meningococcal, herpes zoster, and human papillomavirus vaccines, was < 10% for each. Perceived infection vulnerability was substantial, with 62.7% expressing concern about viral infections due to MS and 71.1% believing that DMTs increased susceptibility, consistent with 59.4% receiving high-efficacy DMTs. Awareness of EBV was limited: 26.5% had heard of EBV, 16.6% perceived an MS–EBV association, and 21.1% were aware of possible causality. Nearly half of participants (49.0%) were undecided about a hypothetical EBV vaccine, while approximately 44% expressed willingness to receive it. Counseling gaps were evident, as only 32.8% reported any discussion about vaccination with clinicians; counseling prior to DMT initiation and during ongoing treatment was reported by 16.2% and 19.1%, respectively.

**Conclusions:**

In a young and highly educated cohort of pwMS in Saudi Arabia, vaccine hesitancy and counseling gaps persist despite high perceived infection risk. These findings highlight the need for structured and routine vaccine counseling, particularly at MS diagnosis and before initiating high-efficacy DMTs, to improve uptake of influenza and other recommended adult vaccines in MS clinics.

## 1 Introduction

Multiple sclerosis (MS) is a chronic, immune-mediated and neurodegenerative disorder of the central nervous system in which adaptive immune cells (CD4+ and CD8+ T cells and B cells) together with innate immune cells (including monocytes/macrophages and activated microglia) drive focal inflammatory demyelination alongside progressive axonal and neuronal injury (1, 2). MS occurs more frequently in females than in males, with individuals aged 20–50 years at the highest risk (3, 4). Globally, approximately 2.8 million people are living with MS, corresponding to a prevalence of 35.9 per 100,000 population (5). In Saudi Arabia, recent epidemiologic reports indicate a gradual increase in both prevalence and incidence, with current prevalence estimated at approximately 40 cases per 100,000 population (6). The etiology of MS remains incompletely understood, although accumulating evidence supports an interaction between genetic susceptibility and environmental exposures (7).

Vaccination against vaccine-preventable diseases is a public health priority. People with MS represent a high-risk group because several disease-modifying therapies (DMTs) increase susceptibility to infection and may attenuate vaccine-induced immune responses. Accordingly, clinical guidelines recommend that pwMS remain up-to-date with routine vaccinations, particularly at the time of diagnosis, and that immunization timing be carefully coordinated with specific DMT regimens (8). Infections are also associated with an increased risk of relapse and may lead to transient neurologic worsening during febrile illness, further emphasizing the importance of preventing avoidable infections through vaccination (9).

Vaccine hesitancy is prevalent in the general population. The World Health Organization Strategic Advisory Group of Experts on Immunization defines vaccine hesitancy as a delay in acceptance or refusal of vaccination despite availability of vaccination services, influenced by the “3Cs” model: confidence, complacency, and convenience (10).

Globally, data on vaccine hesitancy among pwMS are heterogeneous and largely focused on COVID-19 vaccination. Willingness to receive COVID-19 vaccines among pwMS has been reported as high in some settings, including 94.4% in the United Kingdom MS Register, approximately 77% in a United States survey, and similarly low hesitancy in Swiss registry data (11–13). These figures should be compared cautiously: willingness and hesitancy are distinct constructs, measured with different instruments, definitions, and response categories across studies, so cross-study comparisons are indicative rather than exact.

In contrast, a 2021 survey from Ireland reported vaccine hesitancy rates ranging from 10% to 20% among patients with MS (14). Data from Saudi Arabia remain limited; however, regional studies from the Middle East indicate substantial hesitancy, with one Egyptian cohort reporting that only half of patients received the COVID-19 vaccine, primarily due to fear of adverse effects (15, 16).

Vaccine hesitancy among pwMS appears to result from multiple factors, including limited vaccine-related knowledge, misconceptions regarding safety and efficacy, and insufficient vaccine counseling by healthcare professionals involved in MS care. In parallel, evidence consistently demonstrates that patients with MS have a higher baseline risk of infection compared with the general population. This risk increases with progressive disease course and greater disability, and is further amplified by certain DMT classes, such as anti-CD20 therapies and sphingosine-1-phosphate modulators, which are associated with higher or specific infection risks. Notably, exposure to anti-CD20 agents has been linked to worse COVID-19 outcomes in large international registries (17–21).

In addition, converging epidemiologic evidence supports a central role of Epstein–Barr virus (EBV) in MS pathophysiology. Large longitudinal studies indicate that EBV infection precedes nearly all MS cases and is associated with a markedly increased risk of disease development (22, 23). Together, these clinical, behavioral, and virologic considerations underscore the importance of evaluating both vaccination readiness and perceptions of infection-related risk in MS care.

To our knowledge, this is among the first studies from Saudi Arabia to jointly characterize, in people with MS (pwMS), (i) vaccination readiness and uptake, (ii) the clinician-counseling gap around DMT initiation, and (iii) awareness of the Epstein–Barr virus (EBV)–MS association, identifying a modifiable counseling gap—only 32.8% of pwMS reported ever discussing vaccination with a clinician—in a group at elevated infection risk from immunomodulatory therapy.

Therefore, this study aimed (i) to assess patients' readiness to receive vaccination and determine whether they had received counseling or support intended to enhance that readiness, and (ii) to evaluate patients' perceptions of infection-related risk, specifically (a) fear of viral infection attributable to MS, (b) fear of infection related to DMT-associated immunosuppression, and (c) awareness of the potential role of EBV in MS pathophysiology.

## 2 Methods

This study was approved by the Institutional Review Board (IRB) of King Abdulaziz University Hospital (KAUH) (Reference No. 637-23). In July 2024, a cross-sectional study was conducted in Jeddah, Saudi Arabia, targeting pwMS. A target sample size of approximately 200 participants was considered sufficient to estimate proportions with a margin of error of ±6–7% at a 95% confidence level. Given the limited number of patients served by MS clinics in this setting, a non-probability (convenience) approach was used, combining consecutive invitation of eligible patients attending the KAUH MS clinic with outreach through community-based MS support groups, resulting in a final sample of 204 participants. The recruitment source was not recorded at the individual-participant level; therefore, the number of participants recruited through each channel could not be determined retrospectively. Inclusion criteria included a confirmed diagnosis of MS and age ≥18 years. The questionnaire was distributed electronically to patients attending the MS clinic at KAUH in Jeddah. Additional recruitment was conducted through community-based support group databases, including the WASM Society for MS. For participants recruited at KAUH, the MS diagnosis was verified from medical records (physician-confirmed diagnosis per the McDonald criteria); for those recruited through community and support-group databases, MS status was based on self-reported physician diagnosis and was not independently verified.

Data were collected using an anonymous, self-administered electronic questionnaire. Invitations were distributed to patients attending the KAUH MS clinic and to members of the WASM MS Society community database. A total of 204 eligible participants completed the questionnaire and were included in the analysis. Because the questionnaire was distributed through both clinical and community-based channels, including social media, the exact number of individuals who received or viewed the invitation could not be determined; therefore, a conventional response rate could not be calculated. Participation was voluntary and responses were anonymous. Microsoft Excel 2021 was used only for data export and entry, not for data collection. Statistical analyses were conducted using IBM SPSS Statistics version 21. The full questionnaire (English and Arabic versions) is provided as Supplementary material.

### 2.1 Validity and reliability analysis

The questionnaire items assessed perceptions of viral effects and vaccination among patients with MS and were adapted from previously published studies (24–26). A pilot study involving nineteen individuals was conducted to evaluate validity and reliability. Content validity was assessed by an independent panel of three consultant neurologists with expertise in multiple sclerosis: Dr. Alaa Alotaibi (Consultant Neurologist, King Abdulaziz University), Dr. Foziah Alshamrani (Assistant Professor of Neurology, Imam Abdulrahman Bin Faisal University), and Dr. Hind Alnajashi (Associate Professor of Neurology, King Abdulaziz University). The first two experts were independent of the study; Dr. Hind Alnajashi was an author of the study. Each reviewer assessed every item for relevance, clarity, and appropriateness. Items were rated on a 4-point scale, with scores of 1 indicating inadequate, 2 indicating the need for major revision, 3 indicating adequate with minor revision, and 4 indicating adequate, clear, and relevant. The content validity index (CVI) was calculated as the proportion of items rated 3 or 4, with values ≥80% considered indicative of strong validity. The final questionnaire demonstrated a CVI of 94%.

### 2.2 Reliability analysis

A Cronbach's alpha of 0.72 was obtained for the developed questionnaire, indicating acceptable internal consistency (27). Categorical variables were summarized using frequencies and percentages, while continuous variables were presented as means and standard deviations. Responses to Likert-scale items were treated as ordinal data and described using frequency distributions. Missing data were not imputed. No statistical comparisons between subgroups were performed, as the primary objective of the study was descriptive.

Outcome definitions. All outcomes were defined at the level of individual questionnaire items; no composite indices or cut-off scores were computed. Perceived infection vulnerability was captured by the items in Table 1; EBV awareness and knowledge sources by the items in Table 2; vaccination intention by a single 5-point item (“How likely are you to receive routine vaccinations?”); perceived vaccine-related harm by the item on concern that routine vaccines might be harmful; vaccine uptake by self-reported receipt of specific vaccines; and counseling by the items on whether vaccination had been discussed or recommended by a clinician. We did not administer a validated vaccine-hesitancy scale. We therefore report the relevant dimensions separately—vaccination intention, perceived vaccine-related harm, and actual uptake—and we do not classify neutral or undecided responses as hesitancy. Where the term “hesitancy” is retained, it is used descriptively to denote low stated intention to vaccinate and/or endorsement of perceived vaccine harm, not a scored construct.

**Table 1:** Participants' perceptions of the effects of the virus (*n* = 204).

Perceptions of the effects of the virus	Strongly agree	Agree somewhat	I cannot decide	Disagree	Strongly disagree
Being diagnosed with MS, I am very worried about becoming infected with contagious viruses.	68 (33.3)	60 (29.4)	31 (15.2)	25 (12.3)	20 (9.8)
Because I am using disease-modifying therapy, my immune system is very weak and susceptible to viral infections.	76 (37.3)	69 (33.8)	32 (15.7)	20 (9.8)	7 (3.4)
One of the possible causes of MS is a previous viral infection. Do you agree with this statement?	25 (12.3)	42 (20.6)	77 (37.7)	41 (20.1)	19 (9.3)

MS, multiple sclerosis.

**Table 2:** Participants' awareness and perceptions related to EBV (*n* = 204).

Variable	*n* (%)
**Have you heard about EBV before?**	
No	150 (73.5)
Yes	54 (26.5)
**Do you think there is a connection between MS and EBV?**	
Strongly agree	8 (3.9)
Agree somewhat	26 (12.7)
I cannot decide	155 (76)
Disagree	11 (5.4)
Strongly disagree	4 (2)
**Are you aware that there are studies suggesting EBV may be a potential cause of MS?**	
No	68 (33.3)
Not sure	93 (45.6)
Yes	43 (21.1)
**How did you learn about the possible association between EBV and MS)?**	
Through medical professionals	19 (9.3)
Through reading scientific studies	20 (9.8)
Through social media (Twitter, YouTube, TikTok, etc.)	27 (13.2)
I was not aware of the possible association between EBV and MS	138 (67.6)
**If a vaccine for EBV became available, would you be willing to take it and recommend it to others?**	
Strongly agree	53 (26)
Agree somewhat	37 (18.1)
I cannot decide	100 (49)
Disagree	9 (4.4)
Strongly disagree	(2.5)

EBV, Epstein–Barr virus; MS, multiple sclerosis.

## 3 Results

Among the 204 participants, 38.2% were aged 25–34 years, with a mean age of 35.7 years. Females accounted for 57.8% of the cohort, and 66.7% held a bachelor's degree. A diagnosis of MS within the previous 5 years was reported by 39.7% of participants. Most participants (83.3%) were receiving disease-modifying therapy, with high-efficacy intravenous agents (rituximab, ocrelizumab, ofatumumab, and natalizumab) being the most commonly used treatments (59.4%). Regarding motor disability, the majority reported no use of mobility aids (72.5%), whereas 9.3% used wheelchairs, 5.9% consistently used a walking stick or crutch, and 12.3% reported occasional use, as shown in Table 3.

**Table 3:** Distribution of studied participants according to their demographic characteristics and MS clinical data (*n* = 204).

Variable	*n* (%)
Demographic characteristics	
**Age (years)**	
18–24	19 (9.3)
25–34	78 (38.2)
35–44	75 (36.8)
45–54	28 (13.7)
≥55	4 (2)
**Sex**	
Female	118 (57.8)
Male	86 (42.2)
**Education level**	
Does not read or write	1 (0.5)
Reads and writes	4 (2)
High School	49 (24)
Bachelor's Degree	136 (66.7)
Postgraduate	14 (6.9)
Multiple sclerosis clinical data	
**How long have you been diagnosed with MS?**	
< 1 year	16 (7.8)
1–5 years	81 (39.7)
6–10 years	49 (24)
11–15 years	30 (14.7)
>15 years	28 (13.7)
**Are you currently using any treatments for MS?**	
No	34 (16.7)
Yes	170 (83.3)
**If yes, specify: (*****n*** **=** **170)**	
I use one of the following treatments (interferon, dimethyl fumarate, teriflunomide) low efficacy therapy	38 (22.4)
I use one of the following treatments (fingolmoid, cladrbine, sipnmoid) moderate-efficacy oral therapy	24 (14.1)
I use one of the following treatments (rituximab, Ocrevus, ofatumumab, Tysabri) high-efficacy therapy	101 (59.4)
Other treatments	11 (6.5)
**Do you use any mobility aids?**	
No	148 (72.5)
Yes, a walking stick or crutch occasionally	25 (12.3)
Yes, a walking stick or crutch always	12 (5.9)
Yes, a wheelchair	19 (9.3)

MS, multiple sclerosis.

Concerns regarding infectious diseases among pwMS are summarized in Table 1. Overall, 62.7% of participants expressed concern that MS increases susceptibility to viral infections, and 71.1% believed that their disease-modifying therapy further elevated infection risk. Additionally, 32.9% agreed that prior infection with a contagious virus could be associated with an increased risk of developing MS.

Awareness and perceptions of EBV were limited. As shown in Table 2, only 26.5% of participants had heard of EBV, and 16.6% believed that a link exists between MS and EBV. Approximately 21% were aware of studies suggesting EBV as a potential causal factor in MS. Social media platforms, including X (formerly Twitter), YouTube, and TikTok, were the most frequently reported information sources (13.2%), whereas medical professionals accounted for 9.3%. Nearly half of participants (49.0%) were undecided regarding their willingness to receive an EBV vaccine if it became available (Figure 1).

**Figure 1:**
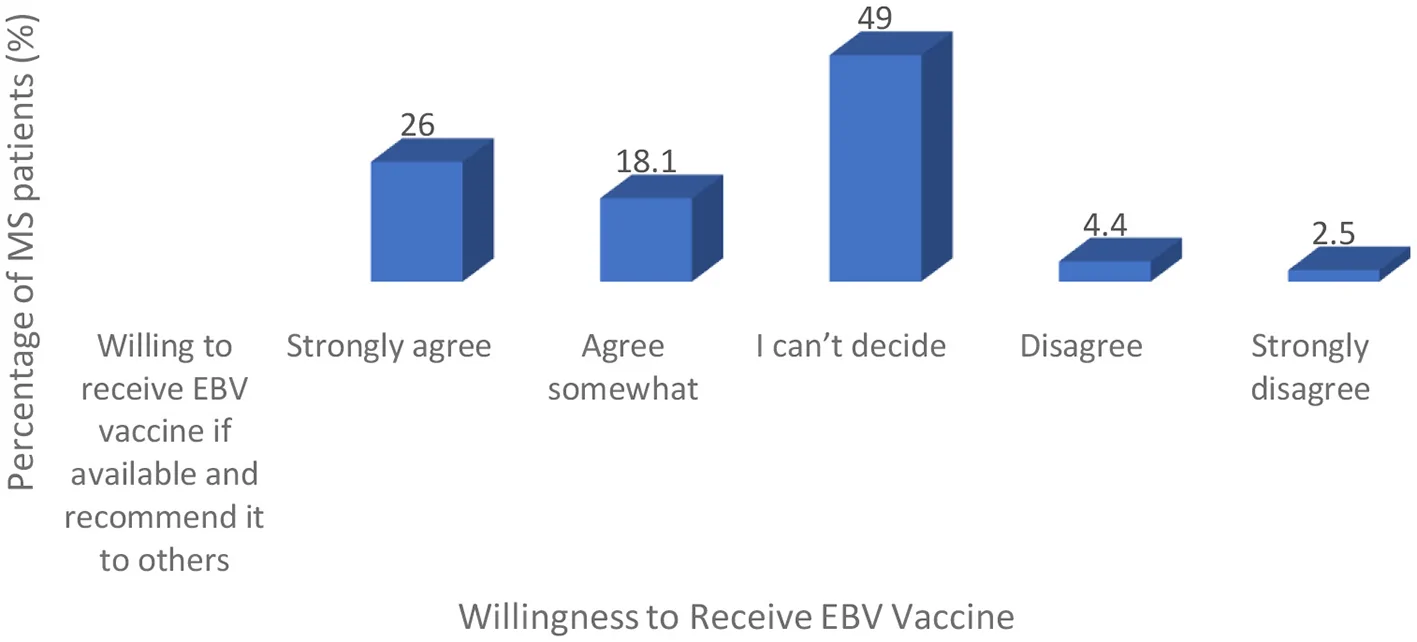
Percentage distribution of pwMS willing to receive an Epstein–Barr virus (EBV) vaccine, if available, and to recommend it to others.

Participants' attitudes toward routine vaccination are presented in Table 4. Regarding intention, 47.6% reported being somewhat or very likely to receive routine vaccines, 33.8% were neutral, and 18.6% were somewhat or very unlikely to do so. Separately, a majority (60.8%) agreed or somewhat agreed that routine vaccines might be harmful for people with MS. These dimensions are reported separately and are not collapsed into a single “hesitancy” figure. Only 32.8% reported having discussed vaccination with their physicians. Among these, 16.2% indicated that vaccination was recommended before initiating MS treatment, while 19.1% received recommendations during ongoing therapy.

**Table 4:** Participants' perceptions related to vaccinations in general and seasonal influenza vaccine specifically (*n* = 204).

Variable	*n* (%)
Perception of vaccinations	
**How likely are you to receive routine vaccinations against viruses?**	
Very likely	44 (21.6)
Somewhat likely	53 (26)
Neutral	69 (33.8)
Somewhat unlikely	26 (12.7)
Very unlikely	12 (5.9)
**As someone with MS, do you have concerns that routine vaccinations might harm you?**	
Strongly agree	56 (27.5)
Agree somewhat	68 (33.3)
I cannot decide	43 (21.1)
Disagree	23 (11.3)
Strongly disagree	14 (6.9)
**Have you had any discussions with your doctor about vaccinations?**	
No	108 (52.9)
I do not remember	29 (14.2)
Yes	67 (32.8)
**Did your treating physician recommend any vaccinations before initiating your treatment for MS?**	
No	130 (63.7)
I do not remember	41 (20.1)
Yes	33 (16.2)
**Did your treating physician recommend any vaccinations during your treatment for MS?**	
No	126 (61.8)
I do not remember	39 (19.1)
Yes	39 (19.1)
Seasonal influenza vaccine	
**Have you received the annual influenza (flu) vaccine this year?**	
No	142 (69.6)
Yes	62 (30.4)
**Individuals with MS are advised to receive the flu vaccine, as it helps protect against complications and is not associated with serious side effects**	
Strongly agree	40 (19.6)
Agree somewhat	49 (24)
I cannot decide	86 (42.2)
Disagree	17 (8.3)
Strongly disagree	12 (5.9)

MS, multiple sclerosis.

Regarding vaccine uptake, 30.9% reported receiving the influenza vaccine during the current year, and 43.6% agreed that pwMS are advised to receive it due to protective benefits and lack of serious adverse effects. As illustrated in Figure 2, the COVID-19 vaccine was the most commonly received vaccine (72.5%), followed by the influenza vaccine (30.9%). Participants expressed a need for additional education on vaccine-related topics, including which vaccines are recommended during MS treatment (41.2%), potential vaccine side effects (40.7%), and vaccines recommended prior to initiating MS therapies (39.7%), as detailed in Table 5.

**Figure 2:**
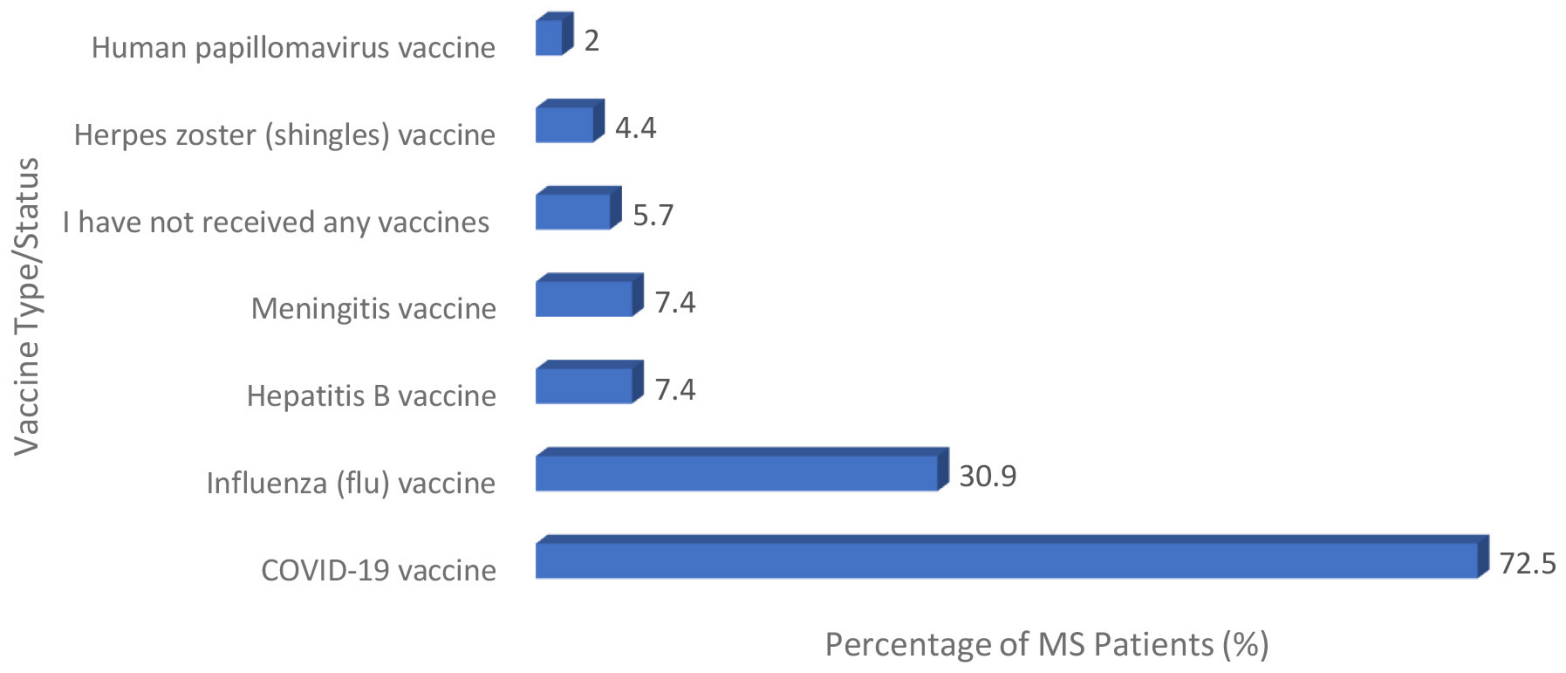
Vaccines received by participants after being diagnosed with multiple sclerosis (MS).

**Table 5:** Vaccines received after being diagnosed with MS and preferred vaccination-related topics participants would like to learn about (*n* = 204).

Variable	*n* (%)
**Have you received any of the following vaccines after being diagnosed with multiple sclerosis?**	
COVID-19 vaccine	148 (72.5)
Influenza (flu) vaccine	63 (30.9)
Hepatitis B vaccine	15 (7.4)
Meningitis vaccine	15 (7.4)
Herpes zoster (shingles) vaccine	9 (4.4)
Human papillomavirus vaccine	4 (2)
I have not received any vaccines	32 (15.7)
**Are there any vaccination-related topics you would like to learn more about?**	
I already have enough information	25 (12.3)
I would like to know which vaccines are recommended before starting MS treatments	81 (39.7)
I would like to know which vaccines are recommended during MS treatment	84 (41.2)
I would like to understand the possible side effects	83 (40.7)
I would like to know if any vaccines could potentially cause MS	62 (30.4)
I believe patients with MS should avoid all vaccines	21 (10.3)
I do not have any additional questions at the moment	31 (15.2)

MS, multiple sclerosis.

## 4 Discussion

This study examined perceptions of viral infection risk and attitudes toward vaccination among pwMS in Saudi Arabia. Despite a relatively young and highly educated cohort, with two-thirds aged 25–44 years (25–34 years: 38.2%; 35–44 years: 36.8%) and 66.7% holding a bachelor's degree, vaccine hesitancy remained evident. This finding suggests that hesitancy is not driven by lower educational attainment and is more consistent with determinants described in the World Health Organization Strategic Advisory Group of Experts “3Cs” framework, including confidence, complacency, and convenience, rather than knowledge alone (10). Compared with settings reporting higher vaccine willingness, such as the United Kingdom MS Register with 94.4% acceptance (11), these results point to context-specific gaps across regions. These cross-setting comparisons should be interpreted cautiously, since willingness and hesitancy are measured with different instruments and definitions across studies. Consistent with findings by Nabavi et al. (24), pwMS appeared generally receptive to new vaccines despite limited understanding of vaccine mechanisms or disease-related risks, underscoring a clear opportunity for targeted education by healthcare professionals.

Perceived infection risk was high in this cohort, with 62.7% expressing concern about viral infections related to MS and 71.1% believing that DMTs increase susceptibility. This perception likely reflects treatment patterns, as 59.4% of participants were receiving high-efficacy therapies, including rituximab, ocrelizumab, ofatumumab, and natalizumab. These concerns align with real-world evidence demonstrating increased infection risk associated with certain high-efficacy DMTs, particularly anti-CD20 agents, compared with lower-efficacy therapies (18), as well as international registry data linking anti-CD20 exposure to worse COVID-19 outcomes (19).

General vaccine hesitancy was substantial. Neutral attitudes toward routine vaccination were reported by 33.8% of participants, and 60.8% agreed or somewhat agreed that routine vaccines might be harmful for pwMS. Vaccine uptake varied markedly by type. COVID-19 vaccination coverage was high (72.5%) whereas, influenza vaccination remained low (30.9%), and uptake of other adult vaccines, including hepatitis B, meningitis, herpes zoster, and human papillomavirus vaccines, was < 10%. This pattern likely reflects the strong public health emphasis and mandatory policies surrounding COVID-19 vaccination in Saudi Arabia. In contrast, lower influenza vaccine uptake highlights a missed opportunity for routine prevention, particularly given existing recommendations for pwMS (28). For reference, current guidance recommends that pwMS keep routine immunizations up to date from the time of diagnosis and, specifically: annual inactivated influenza vaccination; pneumococcal vaccination where indicated; hepatitis B vaccination (particularly before anti-CD20 or other immunosuppressive therapy); completion of indicated non-live vaccines; and administration of any needed live vaccines preferably at least 4 weeks before initiating immunosuppressive DMTs, with live vaccines generally avoided during such therapy (8, 28). Measured against these benchmarks, uptake in this cohort was concordant only for COVID-19 and, to a lesser extent, influenza, and fell short for the other recommended adult vaccines.

A key actionable finding was the substantial counseling gap. Only 32.8% of pwMS reported any discussion about vaccination with a clinician, with 16.2% recalling counseling before initiating a disease-modifying therapy (DMT) and 19.1% during ongoing treatment. This gap is modifiable, as consensus and practice guidelines recommend maintaining up-to-date vaccination from the time of diagnosis and providing clear guidance on vaccine selection, including non-live vaccine schedules, live-vaccine precautions, and timing relative to DMT initiation (8, 28). Because immunosuppressive and immunomodulating therapies increase infection risk in pwMS, vaccination represents a critical preventive strategy to improve health outcomes (18). Implementation of a structured MS vaccination checklist, incorporating pre-DMT catch-up vaccination, annual influenza vaccination, pneumococcal vaccination where indicated, timing considerations for anti-CD20 and sphingosine-1-phosphate therapies, and live-vaccine precautions, alongside a concise educational handout or patient portal message, may meaningfully reduce uncertainty and improve uptake among hesitant individuals. Taken together, the low counseling rates alongside patients' reliance on non-official information sources suggest that the gap identified here is primarily one of communication, operating on both the clinician side (infrequent counseling) and the patient side (limited exposure to authoritative, MS-specific information); this is a modifiable target for MS services rather than a fixed characteristic of the population.

Given the growing interest in the relationship between MS and EBV, this study examined patient perceptions and awareness of recent advances in MS pathophysiology and the proposed causal role of prior EBV infection (23). Overall knowledge of EBV was limited: only 26.5% of pwMS had heard of EBV, 16.6% believed that an MS–EBV association exists, and 21.1% were aware of evidence suggesting a potential causal role. As this area of research is relatively recent, definitive conclusions cannot be drawn from patient awareness alone. However, the findings suggest that pwMS primarily obtain information from local news outlets and patient communities rather than international scientific sources.

The low awareness of EBV in this comparatively young and highly educated cohort is itself noteworthy, given that EBV has been implicated in MS for decades and, more recently, established as a near-necessary antecedent of the disease. A likely explanation is that EBV is popularly known only as the cause of infectious mononucleosis rather than as an MS-relevant exposure, and that MS-specific information is not consistently communicated to patients. Notably, our knowledge-source item (Table 2) did not include an official or governmental health-authority source; in many health systems such official pages, together with NGOs and patient organizations such as the WASM MS Society, are the principal reference points, and scientific findings typically reach patients only as filtered through them. The omission of this response option is a limitation, and the proportion of respondents reporting that they had read the primary scientific literature should be interpreted cautiously, as it may reflect science encountered via social media or a degree of social-desirability over-reporting. Finally, the item on willingness to receive a hypothetical EBV vaccine should be read as a measure of general vaccine acceptability rather than a clinically actionable intention: a prophylactic EBV vaccine would need to be administered well before adolescence—plausibly in early childhood—to prevent delayed primary infection, and could not retrospectively undo EBV's contribution once MS is established (29).

These observations have important public health implications. The persistent counseling gap underscores the need for structured and standardized vaccine counseling protocols within MS services, particularly at diagnosis and before initiation of high-efficacy DMTs. Additionally, closer collaboration and better coordination between MS clinics and primary-care providers is essential to facilitate timely assessment of vaccination status, catch-up immunization, and consistent reinforcement of vaccine recommendations. Establishing shared-care vaccination pathways may improve routine adult vaccine uptake and reduce preventable infections in this vulnerable population. Several limitations should be considered when interpreting these findings. First, the use of convenience sampling, with most data obtained from online MS communities and patients identified through KAUH records, may limit representativeness of the broader MS population in Saudi Arabia. Second, the cross-sectional design captured attitudes at a single time point, restricting causal inference and temporal generalizability. Lastly, recall bias and social desirability bias may have influenced self-reported responses, particularly regarding vaccine safety and uptake. Beyond educational attainment, we did not formally measure income or occupation, so inferences about the sample's socio-economic profile should be made cautiously; and because recruitment relied on voluntary participation through a clinic and community groups, individuals with greater interest in MS or vaccination may have been over-represented, and non-response could not be fully characterized; the direction and magnitude of any resulting participation bias are therefore uncertain. Priority should first be given to replicating, validating, and interpreting these findings on vaccine attitudes and the counseling gap in larger and more representative MS samples. Our results are also consistent with the broader pattern of vaccine hesitancy documented in the general Saudi population, in which perceived vaccine harm and reliance on informal information sources are recurrent themes (30, 31), underscoring that general cultural determinants—not MS-specific factors alone—shape vaccination behavior in this setting. Longitudinal work to track how attitudes evolve over time would be a valuable subsequent step.

## 5 Conclusions

In a young and highly educated cohort of pwMS in Saudi Arabia, substantial vaccine hesitancy persisted despite widespread recognition of infection risk associated with MS and its therapies. Although 62.7% expressed concern about viral infections related to MS and 71.1% perceived increased susceptibility due to DMTs, vaccine uptake was largely concentrated on COVID-19 vaccination (72.5%), likely reflecting mandates and intensive public health messaging. In contrast, seasonal influenza vaccination remained low (30.9%), and uptake of other adult vaccines, including hepatitis B, meningococcal, herpes zoster, and human papillomavirus vaccines, was < 10%. Importantly, only 32.8% of participants reported any discussion about vaccination with clinicians, and counseling before DMT initiation (16.2%) or during ongoing treatment (19.1%) was uncommon, revealing a clear and modifiable systems-level gap.

These findings indicate that vaccine hesitancy among pwMS is not primarily driven by educational attainment. Instead, it aligns with determinants described in the World Health Organization Strategic Advisory Group of Experts “3Cs” framework, including confidence, complacency, and convenience. To address the gap between vaccination intent and behavior, MS services should incorporate structured vaccine counseling at the time of diagnosis and reinforce recommendations during follow-up visits.

These findings derive from a non-probability (convenience) sample recruited at a single tertiary center in Jeddah and through MS community groups, and may not represent the wider MS population in Saudi Arabia; they should be regarded as hypothesis-generating and confirmed in larger, multi-center studies.

## Data Availability

The raw data supporting the conclusions of this article will be made available by the authors, without undue reservation.
