# Supplementary material for: Perception of virus effects and vaccination among people with multiple sclerosis in Saudi Arabia
Source: Front Neurol. 2026 Oct 12;17:1816182. doi: 10.3389/fneur.2026.1816182 (PMC13696312; doi:10.3389/fneur.2026.1816182)
Supplement: Supplementary file 1 [file Data_Sheet_1.pdf]

Section 1: Consent

Do you agree to complete this questionnaire?

The collected data will be used for scientific research purposes, while ensuring the confidentiality and privacy of your identity.

• Yes

• No

Are you diagnosed with Multiple Sclerosis (MS)?

• Yes

• No

القسم الأول: الموافقة

هل توافق على إكمال هذا الاستبيان؟
سيتم استخدام البيانات التي يتم جمعها لأغراض البحث العلمي، مع ضمان سرية وخصوصية هويتك.

•نعم
•لا

هل تم تشخيصك بمرض التصلب المتعدد(MS) ؟

•نعم

•لا

section 2 : Demographics

Age

• Under 18 years
• 18–24 years
• 25–34 years
• 35–44 years
• 45–54 years
• Over 55 years

Gender

• Male
• Female

Educational level

- Cannot read or write
- Can read and write
- High School
- Bachelor’s degree
- Post graduate degree

How long ago were you diagnosed with Multiple Sclerosis (MS)?

- Less than 1 year
- 1–5 years
- 6–10 years
- 11–15 years
- More than 15 years

Are you currently using any treatment for Multiple Sclerosis (MS)?
- I use one of these treatments (Interferon, Avonex, Tecfidera, Sclera, Aubagio)
- I use one of these treatments (Gilenya, Fingolimod, Mavenclad, Mayzent)
- I use one of these treatments (Rituximab, Ocrevus, Kesimpta, Tysabri)
- I do not currently use any treatment
- Other treatment

If you are using another treatment, please specify its name (short answer)

Do you use any mobility aids?

- No
- Yes, a cane occasionally
- Yes, a cane regularly
- Yes, a wheelchair

القسم الثاني: البيانات الديموغرافية

العمر

- أقل من 18 سنة
- 18–24 سنة
- 25–34 سنة
- 35–44 سنة
- 45–54 سنة
- أكثر من 55 سنة

الجنس

- ذكر
- أنثى

مستوى التعليم

•لا يقرأ ولا يكتب
•يقرأ ويكتب
•تعليم ثانوي
•بكالوريوس
•تعليم عالٍ

منذ متى تم تشخيص إصابتك بمرض التصلب المتعدد؟

•أقل من سنة
•1–5 سنوات
•6–10 سنوات
•11–15 سنة
•أكثر من 15 سنة

هل تستخدم حاليًا أي علاجات للتصلب المتعدد؟

•أستخدم إحدى هذه العلاجات (إنترفيرون، أفونكس، تكفيديرا، سكليرا، أوباجيو)
•أستخدم إحدى هذه العلاجات (جيلينيا، فينغوليمود، مافينكلاد، مايزنت)
•أستخدم إحدى هذه العلاجات (ريتوكسيماب، أوكريفوس، كيسيمبتا، تيسابري)
•لا أستخدم علاج
•علاجات أخرى

في حال استخدام علاج آخر، فضلًا اذكر اسم العلاج؟

هل تستخدم أي مساعدات في الحركة؟

•لا
•نعم، عصا أو عكاز أحيانًا
•نعم، عصا أو عكاز دائمًا
•نعم، كرسي متحرك

section 3:Perceptions of the Effects of the Virus

As a person with Multiple Sclerosis (MS), I am very concerned about contracting infectious viruses:

• Strongly agree
• Somewhat agree
• Cannot decide
• Disagree
• Strongly disagree

A patient with Multiple Sclerosis uses treatments, which means that their

immunity is very weak and they are susceptible to viral infections:

- Strongly agree
- Somewhat agree
- Cannot decide
- Disagree
- Strongly disagree

One of the possible causes of Multiple Sclerosis is previous infection with infectious viruses. Do you agree with this statement?

- Strongly agree
- Somewhat agree
- Cannot decide
- Disagree
- Strongly disagree

كوني مصابًا بالتصلب المتعدد، أنا قلق جدًا من إصابتي بفيروسات معدية:

•أتفق جدًا
•أتفق نوعًا ما
•لا أستطيع أن أحدد
•لا أتفق
•لا أتفق إطلاقًا

كون المريض المصاب بالتصلب المتعدد يستخدم علاجات، وكونه هذا يعني أن مناعته المصاب ضعيفة جدًا ومعرض للإصابة بالفيروسات:

•أتفق جدًا
•أتفق نوعًا ما
•لا أستطيع أن أحدد
•لا أتفق
•لا أتفق إطلاقًا

من الأسباب المحتملة للإصابة بالتصلب المتعدد (الإصابة بفيروسات معدية في السابق)، هل تتفق مع هذه العبارة؟

•أتفق جدًا
•أتفق نوعًا ما
•لا أستطيع أن أحدد
•لا أتفق
•لا أتفق إطلاقًا

section 4: Epstein-Barr Virus (EBV) Awareness and Perception

Have you heard of Epstein–Barr virus (EBV) before?

• Yes
• No

Do you think there is a connection between multiple sclerosis (MS) and Epstein-Barr Virus (EBV)? ?

• Strongly agree
• Somewhat agree
• Cannot decide
• Disagree
• Strongly disagree

Are you aware that there is research suggesting that Epstein–Barr virus (EBV) may be a potential cause of Multiple Sclerosis (MS)?

• Yes
• No
• Not sure

How did you learn about the possible link between Epstein-Barr Virus (EBV) and multiple sclerosis (MS)?

• Specialist doctors
• Reading research
• Social media (television, radio, Twitter, etc.)
• Friends or family
• I am not aware of the potential association between Epstein–Barr virus and Multiple Sclerosis

If a vaccine against Epstein–Barr virus (EBV) were available, would you take it and recommend it to people close to you?

• Strongly agree
• Somewhat agree
• Cannot decide
• Disagree
• Strongly disagree

القسم الرابع: الوعي والإدراك بشأن فيروس إبشتاين بار(EBV)

هل سمعت عن فيروس إبشتاين بار (EBV) من قبل؟

•نعم
•لا

هل تعتقد أن هناك علاقة بين الإصابة بالتصلب المتعدد وفيروس إبشتاين بار(EBV) ؟

•أتفق بشدة

•أتفق نوعًا ما
•لا أستطيع أن أحدد
•لا أتفق
•لا أتفق بشدة

هل تعلم أن هناك أبحاثًا تشير إلى أن فيروس إبشتاين بار (EBV) يمكن أن يكون سببًا محتملًا لمرض التصلب المتعدد؟

•نعم
•لا
•غير متأكد

كيف أصبحت على علم بالارتباط المحتمل بين فيروس إبشتاين بار (EBV) والتصلب المتعدد؟

•أطباء مختصون
•قراءتي للأبحاث
•وسائل التواصل الاجتماعي (تلفاز، راديو، تويتر، إلخ)
•من الأصدقاء أو العائلة
•أنا لست على علم بالارتباط المحتمل بين فيروس إبشتاين بار والتصلب المتعدد

لو وُجد لقاح ضد فيروس إبشتاين بار(EBV) ، هل سوف تأخذه وتنصح المقربين بأخذه؟

•أتفق بشدة
•أتفق نوعًا ما
•لا أستطيع أن أحدد
•لا أتفق
•لا أتفق بشدة

section 5: perception of vaccinations

How likely are you to receive routine vaccinations against viruses?

• Very likely
• Likely
• Neutral
• Unlikely
• Very unlikely

As a person with Multiple Sclerosis (MS), you have concerns that routine vaccinations may cause harm to you:

• Strongly agree
• Somewhat agree
• Cannot decide
• Disagree
• Strongly disagree

Have you had any discussions with your doctor about vaccinations?

- Yes
- No
- I do not remember

Has your treating physician recommended that you receive any vaccinations before starting treatment for Multiple Sclerosis (MS)?

- Yes
- No
- I do not remember

Has your treating physician recommended that you receive any vaccination during treatment for Multiple Sclerosis (MS)?

- Yes
- No
- I do not remember

القسم الخامس: التصور عن التطعيمات

ما مدى احتمالية حصولك على التطعيمات الروتينية ضد الفيروسات؟

- من المرجح جدًا
- مرجح
- محايد
- غير مرجح
- غير مرجح أبدًا

كونك مصابًا بالتصلب المتعدد، يوجد لديك مخاوف من تسبب التطعيمات الروتينية بالضرر لك:

- أتفق بشدة
- أتفق نوعًا ما
- لا أستطيع أن أحدد
- لا أتفق
- لا أتفق بشدة

هل أجريت أي مناقشات مع طبيبك الخاص حول التطعيمات؟

- نعم
- لا
- لا أتذكر

هل أوصاك طبيبك المعالج بالحصول على أي تطعيمات قبل بداية العلاج بالتصلب المتعدد؟

- نعم
- لا
- لا أتذكر

هل أوصاك طبيبك المعالج بالحصول على أي تطعيم أثناء العلاج بالتصلب المتعدد؟

•نعم
•لا
•لا أتذكر

section 6: Influenza Vaccine

Have you received the annual influenza vaccine?

• Yes
• No

individuals with multiple sclerosis are advised to get the flu vaccine, as it helps protect against complications and is not associated with serious side effects:

• Strongly agree
• Somewhat agree
• Cannot decide
• Disagree
• Strongly disagree

القسم السادس: لقاح الإنفلونزا

هل تلقيت لقاح الإنفلونزا السنوي؟

•نعم
•لا

يُنصح بلقاح الإنفلونزا لمصابي التصلب للحماية من مضاعفات الإنفلونزا دون وجود أضرار شديدة متوقعة للقاح:

•أتفق بشدة
•أتفق نوعًا ما
•لا أستطيع أن أحدد
•لا أتفق
•لا أتفق بشدة

Section 7: Other Vaccinations

Have you received any of the following vaccinations after being diagnosed with the disease?
You may select more than one answer.

• I have not received any vaccine
• Influenza vaccine
• COVID-19 vaccine
• Meningococcal vaccine
• Shingles or varicella (chickenpox) vaccine
• Hepatitis vaccine
• Human papillomavirus (HPV) vaccine

Are there any vaccination-related topics you would like to learn more about?

You may select more than one answer.

• I already have enough information

• I would like to know which vaccines are recommended before starting MS treatments

• I would like to know which vaccines are recommended during MS treatment

• I would like to understand the possible side effects

• I would like to know if any vaccines could potentially worsen MS

• I believe patients with MS should avoid all vaccines

• I don't have any additional questions at the moment

القسم السابع: التطعيمات الأخرى

هل حصلت على أي من التطعيمات التالية بعد التشخيص بالمرض؟
يمكن اختيار أكثر من إجابة.

•لم أحصل على أي لقاح
•لقاح الإنفلونزا
•لقاح كورونا
•لقاح الحمى الشوكية
•لقاح الحزام الناري أو الجدري المائي
•لقاح الكبد الوبائي
•لقاح الورم الحليمي

هل يوجد أي معلومات إضافية تريد أن تعرفها أكثر عن التطعيم؟
يمكن اختيار أكثر من إجابة.

•لدي معلومات كافية
•نعم، أحتاج أن أعرف ما هي التطعيمات الموصى بها قبل استخدام علاجات التصلب المتعدد
•نعم، أحتاج أن أعرف التطعيمات الموصى بأخذها خلال فترة العلاج
•نعم، أحتاج أن أعرف الآثار المحتملة
•نعم، أحتاج أن أعرف إذا كانت التطعيمات مسببة للتصلب المتعدد
•أعتقد أن المريض يجب أن يتجنب جميع التطعيمات
•لا يوجد معلومات أريد أن أعرفها
